# P01.04. Attenuation of neurological dysfunction and brain infarction with a chinese herbal formula in ischemia-reperfusion induced brain injury of mice

**DOI:** 10.1186/1472-6882-12-S1-P4

**Published:** 2012-06-12

**Authors:** F Cheng, X Wang, X Zhong, Y Zhao, Y Lu, X Wang, Q Wang

**Affiliations:** 1 Beijing University of Chinese Medicine, Beijing, China

## Purpose

Stroke is one of the most common diseases in China, especially ischemic stroke. Although there are significant advances in treatment of stroke in Western medicine, traditional Chinese medicine also plays an important role in treatment of patients suffering from acute ischemic stroke in China. One Chinese herbal formulation named Qingkailing solution is derived from an ancient formulation of Angongniuhuangwang and has been documented to reduce ischemic injury of the brain. However, due to significant side effects associated with Qingkailing solution, we reconstituted components of Qingkailing solution and formed a new solution named JZQKL. In the study, we are investigating the effect of different concentrations of JZQKL on ischemia induced brain injury.

## Methods

Mice were employed to induce ischemia injury of brain by middle cerebral artery occlusion (MCAO). JZQKL solution was injected into mice through the tail vein at different times (0, 1.5, 3, 6, and 9 hours after MCAO) and at different doses (0, 1.5, 3, and 6ml/kg). Twenty-four hours after MCAO, neurological function and brain infarction were examined and cell apoptosis at the hippocampus and prefrontal cortex were evaluated by TUNEL, western blot and ROS.

## Results

After examining the effect of different concentrations of JZQKL on neurological function and brain infarction, we identified that there was a good correlation between concentrations and improvement of neurological function. With a concentration of 3ml/kg, time dependent experiments were conducted. Improvement of neurological function and reduction of infarction gradually decreased with delay of administration of JZQKL. Moreover, JZQKL reduced apoptosis of neurons in the hippocampus and prefrontal cortex as documented by reduction of caspase-3 expression, eIF2a phosphorylation and caspase-12 activation. JZQKL also decreased oxygen-reactive species (ROS) in the brain.

## Conclusion

Early administration of JZQKL significantly prevented ischemic induced brain injury and its mechanism might be related to an anti-oxidative effect and/or attenuation of apoptosis in the brain.

